# First description of the immature stages of *Dasyheleaalula* and a redescription of adults from China (Diptera, Ceratopogonidae)

**DOI:** 10.3897/zookeys.824.31722

**Published:** 2019-02-14

**Authors:** Chen Duan, Xiao Hong Jiang, Qiong Qiong Chang, Xiao Hui Hou

**Affiliations:** 1 Zunyi Medical University, Zunyi 563000, Guizhou Province, China Zunyi Medical University Zunyi China

**Keywords:** Adult, aquatic, biting midge, China, description, fourth instar larva, pupa

## Abstract

The fourth instar larva and pupa of *Dasyheleaalula* Yu, 2005 are described and illustrated using a Scanning Electron Microscope. The adult male and female of this species are redescribed. Immatures were collected from flooded soil near a pond in Xiaojiawan village, Guizhou province, China and reared in the laboratory. The studied material is deposited in the Insect Collection of Zunyi Medical University.

## Introduction

Biting midges of the genus *Dasyhelea* Kieffer, 1911 (Diptera: Ceratopogonidae) are common and widespread, and are found in all regions of the world in a wide variety of habitats (Wirth and Linley 1990). At present there are 191 species of the genus in China (Yu et al. 2005, 2006, 2015; Lai et al. 2006, 2015, 2016, 2018; Yan and Yu 2008; Sun et al. 2010; Nie et al. 2016; Yang et al. 2017; Mahe et al. 2018), but only seven of these species are known as their immature stages (Yu et al. 2005, 2013). *Dasyheleaalula* Yu, 2005 belongs to the subgenusDasyhelea (*Pseudoculicoides* Malloch), 1915 and *johannseni* group as defined by Dominiak (2012) and Szadziewski (1985). The purpose of this paper is to provide a complete description, with illustrations, of the fourth instar larva and pupa of *Dasyheleaalula* and a redescription of the adult male and female using a compound microscope and Scanning Electron Microscope.

## Materials and methods

The specimens were collected with the aid of a scoop from flooded soil in Xiaojiawan village, Guizhou province, China and carried to the laboratory in summer of 2018. They were placed in separate Petri dishes (larvae) and glass vials (pupae) with a small amount of water and reared in an environmental chamber (LZX-300L-III, Shanghai Xinlang Electronic Technology Ltd, Shanghai, China) maintaining 28 ± 2 °C temperature, RH 75 ± 2% and photoperiod of 12 h: 12 h (6W LED tube-light). The pupae were observed daily until adult emergence. The emergent adults and whole larvae and pupae were preserved in ethanol. The specimens were mounted in Canada balsam following Yu et al. (2005). For the Scanning Electron Microscope (SEM) study, one larva of *D.alula* was prepared following the technique of Ronderos et al. (2000, 2008). Ink illustrations were made using an attached camera lucida. Photographs of specimens were taken with a digital system adapted to an Olympus BX43 with a digital camera DP26. Studied material was deposited in the Insect Collection, Zunyi Medical University, Guizhou province, China (ICZU). The morphological terms and identification methods used in the study follow Yu et al. (2005), Díaz et al. (2013) and Borkent (2014). The following abbreviations are used:

**HL** head length;

**HW** head width;

**HR** head ratio;

**SGR** ratio of subgenal and head width;

**SGW** subgenal width;

**MDL** mandible length;

**MDW** mandible width;

**LAW** width across the lateral arms of epipharynx;

**DCW** width across each of the paired dorsal comb sclerites of the epipharynx;

**ROL** respiratory organ length;

**ROW** respiratory organ width;

**ROP/ROL** respiratory organ pedicel length / respiratory organ length;

**DAL** dorsal apotome length;

**DAW** dorsal apotome width;

**DAW/DAL** dorsal apotome width / dorsal apotome length.

## Results

### Dasyhelea alula

Taxon classificationAnimaliaDipteraCeratopogonidae

Yu, 2005

Dasyhelea (Pseudoculicoides) alula Yu, 2005: 259 (male and female, China)

#### Material examined.

2 males, 3 females, 3 larvae, 3 larval exuviae, 2 male pupal exuviae, 3 female pupal exuviae. Xiaojiawan village, Xinpu new district, Zunyi city, Guizhou province, China, 27°43'22.83"N; 107°04'27.62"E, 7.VII.2018, alt. 866 m, Chen Duan leg.

#### Descriptions.

**Figure 1.**
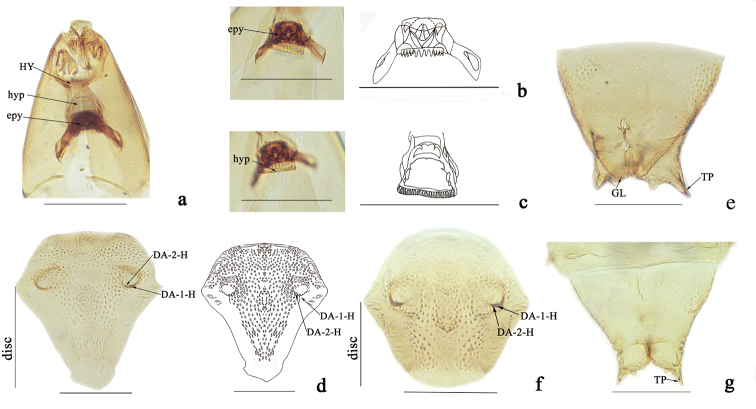
*Dasyheleaalula* Yu. Larva (**a–c**), male pupa (**d–e**), female pupa (**f–g**). **a** head, dorsal view **b** epipharynx, dorsal view **c** hypopharynx, dorsal view **d** dorsal apotome (male) **e** segment IX (male) **f** dorsal apotome (female) **g** segment IX (female). Scale bars: 0.1 mm. Abbreviations: hypostoma (**HY**); hypopharynx (**hpy**); epipharynx (**epy**); genital lobe (**GL**); terminal process (**TP**).

**Figure 2.**
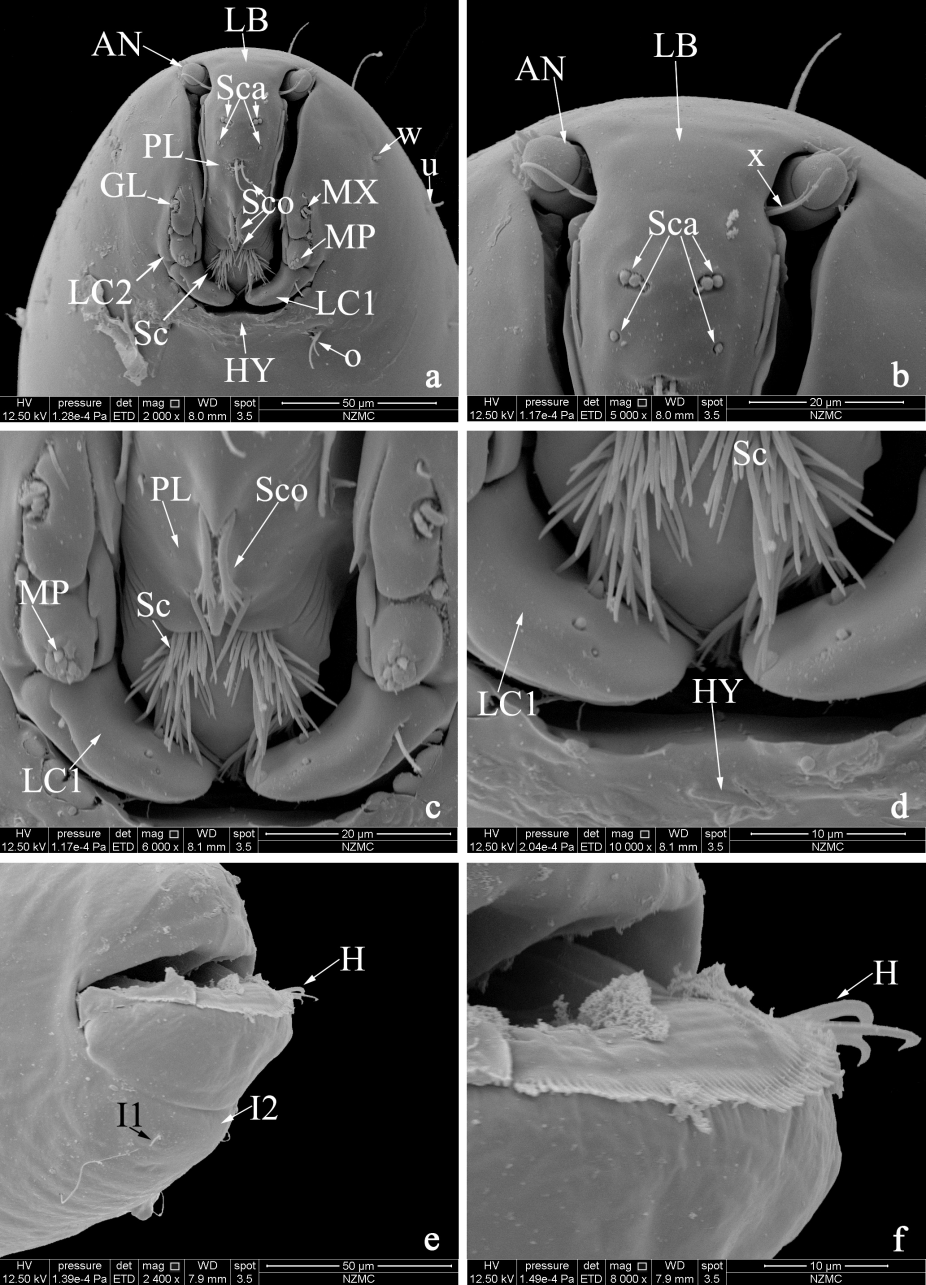
*Dasyheleaalula* Yu, larva**. a** head capsule (palatum, frontal view) **b** detail of labrum **c** detail of scopae **d** detail of lacinial sclerite I **e** caudal segment **f** detail of caudal segment. Abbreviations: antenna (**AN**); galeolacinia (**GL**); hypostoma (**HY**); hooks (**H**); labrum (**LB**); lacinial sclerite I (**LC1**); lacinial sclerite II (**LC2**); maxilla (**MX**); maxillary palpus (**MP**); palatum (**PL**); sensilla coeloconica (**Sco**); sensilla campaniformia (**Sca**); scopae (**Sc**).

**Fourth instar larva (Figs 1a–c; 2a–f).** Head capsule yellowish, short, wide (Fig. 1a); HL 0.20-0.21 (0.20, n = 2) mm; HW 0.15–0.16 (0.15, n = 2) mm; HR 1.31–1.33 (1.32, n = 2); SGW 0.07–0.08 (0.08, n = 2) mm; SGR 0.47–0.50 (0.49, n = 2). Antenna short, cylindrical. Anterior portion of palatum (Figs 1a, 2a, b) with four pairs of campaniformia sensilla, posterior portion with three pairs of coeloconica sensilla, two simple, one serrate (Fig. 2a, c); messors well developed; scopae (Fig. 2a, c, d) well developed with more than 67 elongate, strong pointed teeth; maxilla (Fig. 2a) well sclerotized; mandible (Fig. 1a) stout, with three similar teeth, MDL 0.06 mm, MDW 0.02 mm; galeolacinia (Fig. 2a) with concentrated flap-like papillae, one seta; maxillary palpus (Fig. 2a, c) cylindrical, with 5–6 apical papillae; lacinial sclerite I with one short seta, dorsal view of lacinial sclerite I with three pairs of lobes; lacinial sclerite II without seta. Hypostoma (Fig. 2a) with mesal portion smooth, flanked with four strong, lanceolate lateral teeth on each side. Epipharynx (Fig. 1b) massive, strongly sclerotized, dorsal comb moderately wide, round, with 12 teeth, subequal elongate, lateral arms stout, elongate, with two auxiliary sclerites. LAW 0.08–0.13 (0.11, n = 3), DCW 0.03–0.04 (0.04, n = 3). Hypopharynx (Fig. 1c) stout, heavily sclerotized, posterior comb straight with fringe, with labium sclerotized. Thoracic pigmentation diffused, pale brown. Abdominal segments whitish, with diffused pale brown pigmentation. Caudal segment (Fig. 2e, f) with single comb, which has dense elongate and subequal teeth, with three long and stout crooked hooks.

**Figure 3.**
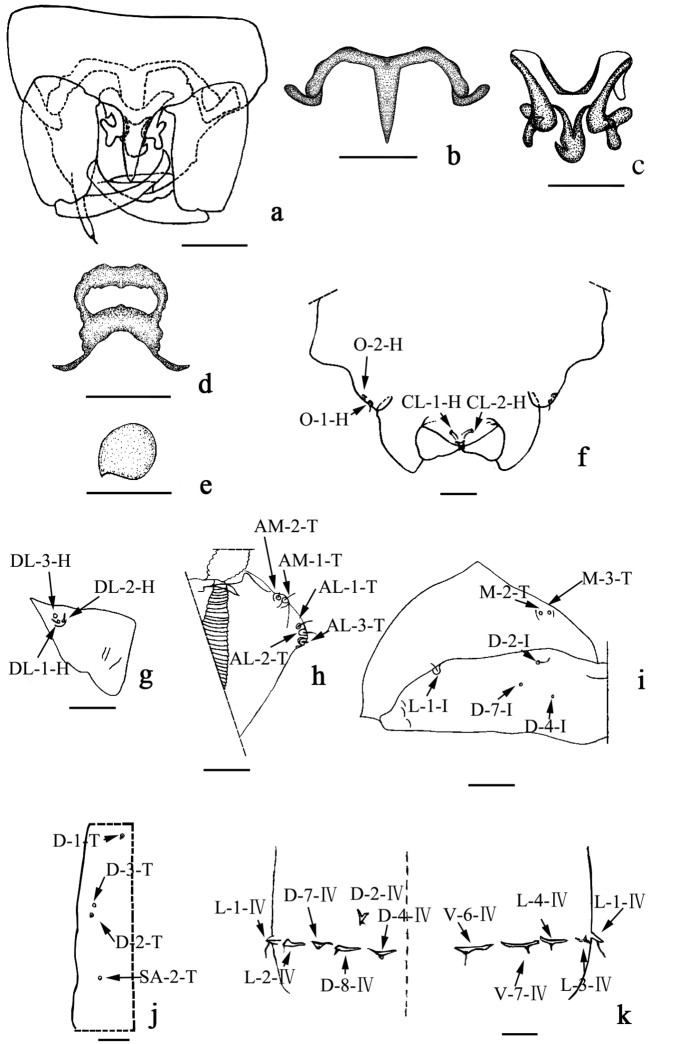
*Dasyheleaalula* Yu. Male adult (**a–c**), female adult (**d–e**), male pupa(**f–k**). **a** genitalia **b** parameres **c** aedeagus **d** subgenital plate **e** spermatheca **f** clypeal/labral sensilla and ocular sensilla **g** dorsolateral cephalic sclerite sensilla **h** anterolateral and anteromedial sensilla **i** metathoracics sensilla, lateral and dorsal sensilla of first abdominal segment **j** dorsal and supraalar sensilla **k** dorsal, lateral and ventral sensilla of segment IV. Scale bars: 0.1 mm.

**Pupa (Figs 1d–g; 3f–k). *Male.*** Total length 1.97 mm. General coloration of exuviae pale brown. Dorsal apotome (Fig. 1d) 2.7 × broader than long, triangular, with apex rounded, surface covered with brown rounded tubercles, anterior margin straight, lateral margin smooth, with three anterior wrinkles; apotome sensilla (Fig. 1d): DA-1-H elongate, thin seta, insert on well-developed tubercle, DA-2-H campaniform sensillum at base; disc surface covered by stout, rounded spinules; DAL 0.07 mm, DAW 0.19 mm, DAW/DAL 2.71. Respiratory organ, apex medium dark brown, 7.5 × longer than broad, with circular fold, 7–8 apical, three lateral pores; ROL 0.15 mm, ROW 0.02 mm; pedicel pale brown, short, length 0.01 mm, ROP/ROL 0.07. Mouthparts with mandible, lacinia absent; two clypeal/labrals (Fig. 3f), CL-1-H and CL-2-H both medium-sized and thin setae; two ocular sensilla, O-1-H long, thin seta, O-2-H campaniform sensillum (Fig. 3f); cephalothorax surface with small rounded tubercles, length 0.63 mm, width 0.45 mm; cephalothoracic sensilla as follows: three dorsolateral cephalic sclerite sensilla (Fig. 3g), DL-1-H and DL-2-H both medium-sized, thin setae, DL-3-H campaniform sensillum; three anterolateral sensilla (Fig. 3h), AL-1-T, AL-2-T both medium-sized, thin setae, AL-3-T short, stout seta; two anteromedial sensilla (Fig. 3h), AM-1-T medium-sized, thin seta, AM-2-T short, thin seta. Cephalothorax length 0.63 mm, width 0.45 mm. Cephalothoracic sensilla as follows (Fig. 3j): three dorsal setae (D-1-T, D-2-T, and D-3-T), D-1-T and D-2-T short, thin setae, D-3-T campaniform sensillum, SA-2-T supraalar campaniform sensillum. Metathoracic sensilla (Fig. 3i): M-2-T and M-3-T both campaniform sensilla. Abdomen: covered with short, stout spinules on anterior, posterior margin. Tergite I (Fig. 3i) with two depressions in the middle, setae as follows: D-2-I medium-sized, thin seta; D-4-I, D-7-I both campaniform sensilla; L-1-I long, thin seta. Segment IV (Fig. 3k) with sensillar pattern, as follows: D-2-IV short, stout seta; D-4-IV, D-7-IV both campaniform sensilla, D-8-IV short, stout seta, all located on flattened tubercles; L-1-IV long and stout setae, L-2-IV short, thin seta, L-3-IV short, thin seta, L-4-IV short, stout seta, all located on triangular tubercles; V-6-IV long, thin seta, V-7-IV short, stout seta, also located on flattened tubercles. Segment IX (Fig. 1e) 0.95 × longer than wide, length 0.19 mm, width 0.20 mm; ventral and dorsal surface with many spinules; terminal process triangular, elongated, acute, length 0.07 mm.

***Female.*** Similar to male with usual sexual differences. Total length 1.64–1.75 (1.70, n = 2) mm. General coloration of exuviae pale brown, except dorsolateral cephalic sclerite brown. Dorsal apotome (Fig. 1f), DAL 0.08 mm, DAW 0.18 mm, DAW/DAL 2.25. Cephalothorax length 0.71 mm, width 0.42 mm. ROL 0.16–0.18 (0.17, n = 3) mm, ROW 0.02 (n = 3) mm; pedicel length 0.01 (n = 3) mm, ROP/ROL0.11–0.13 (0.12, n = 3). Segment IX (Fig. 1g) length 0.17–0.20 (0.19, n = 2) mm, width 0.20 (0.15, n = 2) mm; ventral surface with many spicules, single funnel-like structure medially. Terminal process (Fig. 1g) triangular, elongated, pointed, length 0.02–0.03 (0.02, n = 2) mm.

#### Redescription of adults (Figs 3a–e; 4a–k).

***Male***

#### (Figs 3a–c; 4a–e).

**Figure 4.**
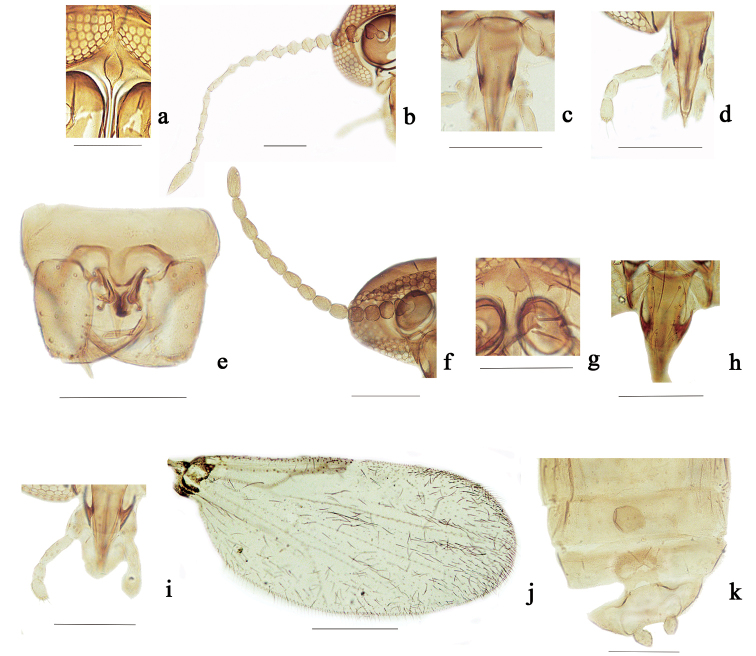
*Dasyheleaalula* Yu. Male adult (**a–e**), female adult (**f–k**). **a** frontal sclerites, anterior view **b** flagellomeres, anterior view **c** clypeus, anterior view **d** palpus, anterior view **e** genitalia, ventral view **f** flagellomeres, anterior view **g** frontal sclerites, anterior view **h** clypeus, anterior view **i** palpus, anterior view **j** wing **k** subgenital plate and spermatheca, ventral view. Scale bars: 0.1 mm.

**Head.** Eyes (Fig. 4a) contiguous, abutting medially for length of 1.0 ommatidia, with interfacetal hairs. Antennal flagellum (Fig. 4b) brown, with distinct sculpture, sparsely plumose, flagellomere 13 without apical projection; AR 1.28. Frontal sclerite nearly round, with long, slender ventral projection (Fig. 4a). Clypeus (Fig. 4c) with four pairs of setae. Palpus (Fig. 4d) brown; third segment slender, the length almost the sum of the fourth and fifth segment. Lengths of palpus segments in ratio of 5: 8: 27: 12: 14.

**Thorax.** Scutum dark brown, scutellum yellow, with six stout setae. Legs brown; hind tibial comb with eight spines; foreleg TR 2.18, midleg TR 2.21, hind leg TR 2.33. Wing length 1.12 mm, width 0.33 mm, CR 0.40; wing membrane hyaline, densely covered with microtrichia, cubital fork at same level of distal portion of second radial cell.

**Abdomen.** Brown. Tergite IX nearly trapezoidal with prominent apicolateral processes. Posteromedial margin of sternite IX with elongate, slender projection, gonostylus slender (Figs 3a, 4e). Parameres fused, with median lobe short, thick, lateral lobe directly ventrolaterally (Figs 3b, 4e). Aedeagus complex, median process thick, long, its lateral processes each with curved apex (Figs 3c, 4e).

***Female* (Figs 3d–e; 4f–k).**

**Head.** Eyes contiguous. Antennal flagellum (Fig. 4f) brown, without sculpture, flagellomere 13 without apical projection; AR 0.93. Frontal sclerite oval, with long, slender ventral projection (Fig. 4g). Clypeus (Fig. 4h) with seven pairs of setae. Palpus (Fig. 4i) brown; third segment slender, without capitate sensillae, lengths of palpus segments in ratio of 4: 7: 17: 7: 12.

**Thorax.** Hind tibial comb with seven spines; foreleg TR 2.00, midleg TR 2.14, hind leg TR 2.16. Wing length 0.82 mm, width 0.34 mm, CR 0.50 (Fig. 4j).

**Abdomen.** Similar to male. Subgenital plate (Figs 3d, 4k) flat, ring-shaped, posterolateral arms sclerotized into darker bands. Spermatheca round (Figs 3e, 4k), strongly pigmented, diameter 53.60 μm, neck short, stout, oblique, length 6.20 μm.

#### Distribution.

**Figure 5.**
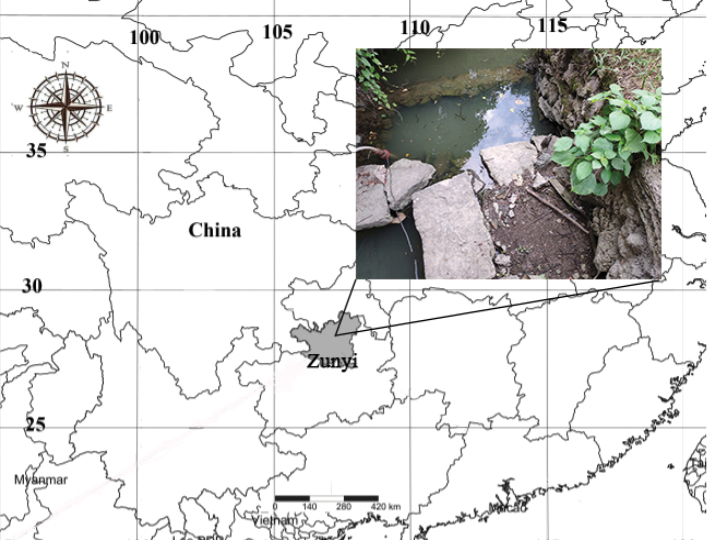
Geographical location of collecting site (inset) of *Dasyheleaalula* in Guizhou province, China.

China (Guizhou province Fig. 5).

## Discussion

*Dasyheleaalula* belongs to the subgenusPseudoculicoides and the *johannseni* group, of which are there 12 species in China: *D.arciforceps* Tokunaga, *D.alula* Yu, *D.curtus* Yu & Yan, *D.communis* Kieffer, *D.ermeri* Remm, *D.excellentis* Borkent, *D.microsporea* Hao & Yu, *D.navai* Xue & Yu, *D.subcommunis* Yu, *D.turficola* Kieffer, *D.turanicola* Remm, and *D.tessicola* Remm. Other than *D.alula*, the larvae and pupae of *D.communis* are the only described immatures of any species within this group. The larva of *D.alula* is similar to *D.communis* by virtue of the mandible with three same-sized teeth, but the dorsal comb of epipharynx has small and dense teeth. In addition, the larva of *D.alula* is also similar to that of *D.mediomunda*, the shared features as follows: head capsule is short, the medial portion of the hypostoma smooth, the lateral arms of the epipharynx stout and lacking teeth, but the larva of *D.mediomunda* differs by having inconspicuous scopae, the mandible with two teeth and the anterior portion of palatum with three pairs of campaniformia. The pupa of *D.alula* is similar to that of *D.eloyi* with scale-like spines on the respiratory organ, but the latter differs by having 16–18 apical and 5–6 lateral pores. The pupa of *D.alula* otherwise matches the generic features of *Dasyhelea* as described by Borkent (2014). The larva and pupa of *D.caeruleus* and an unidentified species of *Dasyhelea* also breed in the small wetland, and the larva and pupa of two species of *Forcipomyia* Meigen were also found in the same place. The small wetland was near a fishpond surrounded by an orchard containing *Prunuscerasifera* Ehrh, *Pyrussorotina* Will, and *Prunuspersica* (L.) Batsch.

## Supplementary Material

XML Treatment for Dasyhelea alula
